# *d*-Band Engineering of Layered (Fe_1−*x*_Ni*_x_*)_3_GaTe_2_ for Enhanced Alkaline Hydrogen Evolution by Ni-Substitutional Doping

**DOI:** 10.3390/nano16130820

**Published:** 2026-07-02

**Authors:** Xiaomin Tian, Yuan Cao, Huilin Zhou, Fanjie Tan, Ziqin Zhang, Liying Pei, Yi Ma, Jianzhi Gao, Wenliang Zhu, Minghu Pan

**Affiliations:** 1 School of Physics and Information Technology, Shaanxi Normal University, Xi’an 710062, China; xiaomintian@snnu.edu.cn (X.T.); cao_yuan@snnu.edu.cn (Y.C.); 20211840@snnu.edu.cn (H.Z.); fanjietan@snnu.edu.cn (F.T.); zhangziqin@snnu.edu.cn (Z.Z.); peiliying@snnu.edu.cn (L.P.); jianzhigao@snnu.edu.cn (J.G.); 2 School of Chemistry and Chemical Engineering, Shaanxi Normal University, Xi’an 710062, China

**Keywords:** alkaline hydrogen evolution reaction, *d*-band center, substitutional-doping, Fe_3_GaTe_2_

## Abstract

Tuning the *d*-band electronic structure of non-noble-metal catalysts is a central strategy for an alkaline hydrogen evolution reaction (HER), yet how composition controls the *d* orbital in multi-Wyckoff-site layered systems remains insufficiently understood. Here, layered (Fe_1-*x*_Ni*_x_*)_3_GaTe_2_ single crystals (*x* = 0.2–1.0) were synthesized by the self-flux method as a platform to address this question. Single-crystal XRD and EDS confirm that Ni is uniformly incorporated into the parent P6_3_/mmc framework while inducing a composition-dependent lattice evolution. Electrochemical measurements in 1.0 M KOH reveal a clear volcano-shaped composition dependence, peaking at *x* = 0.6, where the lowest overpotential, the smallest Tafel slope (94 mV dec^−1^), the lowest charge-transfer resistance and the largest double-layer capacitance are simultaneously reached. First-principles calculations show that Ni doping reshapes the Fe-site *d* orbital strongly composition-dependent rate: the Fe *d*-band center upshifts rapidly by ~0.5 eV between *x* = 0.4 and *x* = 0.6, while the Ni *d*-band center stays nearly fixed in the same composition range. The maximum of HER activity therefore aligns with a steep upshift of the Fe *d*-band center rather than with the Ni content itself. Charge-density mapping of (Fe_0.4_Ni_0.6_)_3_GaTe_2_ further demonstrates that the electron-enriched regions are located on the Fe and interlayer Ni3 sublattices that dominate the *d* states near E_F_.

## 1. Introduction

Hydrogen is widely regarded as a promising energy carrier for future sustainable energy systems because of its high energy density [1] and environmentally benign combustion products [2]. Among various hydrogen-production technologies, water electrolysis is one of the most attractive approaches, in which the hydrogen evolution reaction (HER) plays a central role [3]. Although Pt-based catalysts exhibit reasonable HER activity, the scarcity and high cost of Pt-based catalysts severely limit large-scale practical application [4]. Recent advances in single-atom Pt catalysts on carbide supports have demonstrated a significant reduction in Pt loading while maintaining high HER activity in alkaline media [5].

Among non-noble-metal candidates, layered transition-metal chalcogenides have attracted broad interest due to their tunable composition, electronic states, anisotropic structures, and accessible surface sites [6,7,8,9]. Their activity can be tuned via heteroatom substitution [10], phase engineering [11], and interfacial electronic coupling [12]. Beyond conventional non-magnetic chalcogenides, the magnetic ordering of layered ferromagnets has recently been recognized as an additional handle for tuning interfacial electron transfer and adsorption energetics in HERs and OERs [13,14], yet how composition controls activity in such systems remains underexplored. For instance, phase transformation in Mo-based perovskites has been shown to regulate metal valence states and oxygen vacancies, thereby enhancing alkaline HER kinetics [15]. Previous studies have shown that for alkaline HERs, the reaction kinetics depend not only on hydrogen-binding strength but also on interfacial electron transfer and water dissociation, all of which are governed by the *d*-band character of transition-metal sites [16]. In particular, alkaline HERs on bimetallic systems often benefit from a dual-site mechanism in which one metal facilitates water dissociation while the other binds and recombines H* [17,18], motivating the present systematic exploration of Fe/Ni partitioning across distinct Wyckoff sites in (Fe_1-*x*_Ni*_x_*)_3_GaTe_2_.

**Figure 1:**
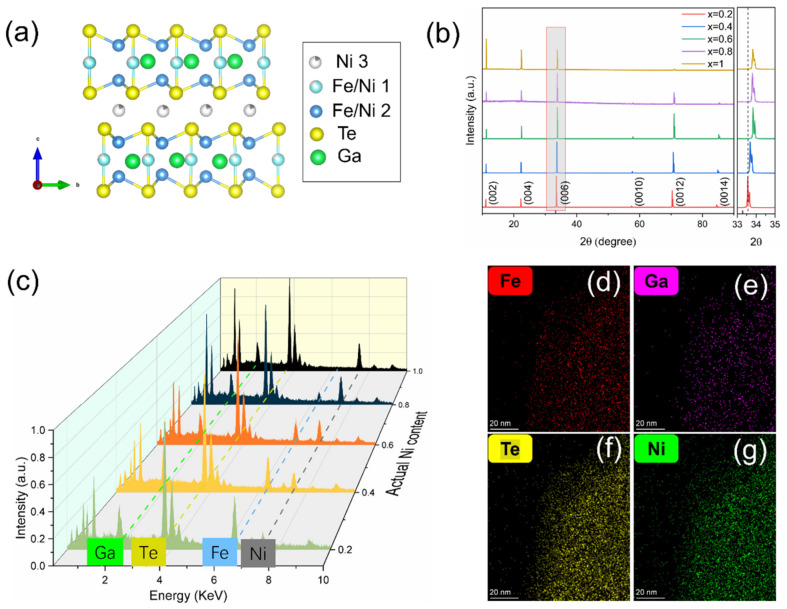
**Structural and compositional characterization of (Fe_1-*x*_Ni*_x_*)_3_GaTe_2_.** (**a**) Schematic crystal structure of (Fe_1-*x*_Ni*_x_*)_3_GaTe_2_ viewed along the a-axis, showing the three transition-metal Wyckoff sites (interlayer Ni3, intralayer Fe/Ni2 and Fe/Ni1) together with the Ga and Te sublattices. (**b**) Single-crystal XRD patterns of samples with different Ni contents, with the (00*L*) reflections indexed (*l* = 2, 4, 6, 10, 12, 14); the inset shows an enlarged view of the (006) peak in the 31–35° range, highlighting the composition-dependent peak shift induced by Ni doping. (**c**) EDS spectra of samples with different Ni contents, showing the characteristic Fe, Ga, Te and Ni signals; the actual Ni content increases monotonically with the nominal composition. (**d**–**g**) EDS elemental mappings of the *x* = 0.6 sample for Fe, Ga, Te and Ni, respectively, revealing uniform elemental distribution without segregation.

Layered magnetic tellurides provide a suitable platform for such an investigation. Compared with many binary chalcogenides, multicomponent tellurides possess a relatively extended *d*-band character compared with binary chalcogenides and more flexible local coordination environments, which make their electronic properties highly sensitive to element substitution [19,20,21,22]. In particular, (Fe_1-*x*_Ni*_x_*)_3_GaTe_2_ crystallizes in a van der Waals layered hexagonal structure (space group P6_3_/mmc), in which Fe atoms occupy two inequivalent Wyckoff sites (Fe1 and Fe2), while Ga and Te form their own sublattices. Several recent studies have demonstrated that layered Fe-based magnetic tellurides such as Fe_3_GeTe_2_ exhibit promising electrocatalytic activity toward hydrogen and oxygen evolution [23], while a recent comparison between Fe_3_GaTe_2_ and Fe_3_GeTe_2_ further revealed that the catalytic activity of these structurally homologous materials originates predominantly from Fe-derived *d* orbitals [24]. These findings establish (Fe,Ni)_3_GaTe_2_ as a particularly suitable platform for studying the *d*-band engineering of HER activity in multi-Wyckoff-site layered magnets. Recent atomic-scale work has further revealed that Ni substitution in this system follows a well-defined three-step occupancy road map: Ni atoms first fill the interlayer Ni_3_ sites in the vdW gap (absent in the parent Fe_3_GaTe_2_) at low doping levels, then progressively occupy the Fe2 sites, and finally substitute the Fe1 sites at a high Ni content [19], indicating a composition-dependent local electronic-structure reconstruction (Figure 1a).

The above studies have established the structural and electronic tunability of (Fe_1-*x*_Ni*_x_*)_3_GaTe_2_. However, how Ni-driven *d*-band evolution affects HER kinetics remains unclear. In particular, how the preferential occupancy of Ni at different Wyckoff sites influences the catalytic performance remains unclear. To address this question, it is essential to first establish a reliable kinetic framework for evaluating the HER activity. In this regard, exchange current density and overpotential are related via the Butler–Volmer equation. Yet they are influenced by different factors, such as the transfer coefficient, surface coverage, and interfacial electric field [25,26,27]. Therefore, a single parameter cannot fully capture the catalytic picture. Recent work also shows that the rate constant $k_{0}$ in the Norskov model is not universal [28]. Instead, $k_{0}$ scales exponentially with hydrogen adsorption free energy (${\Delta G}_{H^{*}}$). This underscores the critical role of electronic structure in determining HER kinetics.

Motivated by these insights, we systematically investigate how Ni-substitutional doping reshapes the alkaline HER behavior of layered (Fe_1-*x*_Ni*_x_*)_3_GaTe_2_. By combining electrochemical measurements with first-principles calculations—projected density of states, *d*-band-center extraction, and charge-density mapping—we trace the composition-dependent activity to a site-projected electronic effect of Ni on the Fe sublattice. The (Fe_1-*x*_Ni*_x_*)_3_GaTe_2_ system thereby offers a platform for connecting dopant occupancy, *d*-band evolution, and HER activity in a multi-Wyckoff-site layered magnet.

## 2. Materials and Methods

### 2.1. Sample Preparation

A series of (Fe_1−*x*_Ni*_x_*)_3_GaTe_2_ samples (*x* = 0.2, 0.4, 0.6, 0.8, and 1.0) were prepared by a self-flux method. High-purity Fe powder (Aladdin, Shanghai, China, 99.9%), Ni powder (Alfa Aesar, Shanghai, China, 99.8%), Ga (Aladdin, Shanghai, China, 99.99%), and Te powder (Alfa Aesar, Shanghai, China, 99.999%) were weighed according to the nominal atomic ratio of (Fe + Ni):Ga:Te = 1:1:2, with the Fe:Ni ratio adjusted to achieve the desired Ni content (*x* = Ni/(Fe + Ni)). The raw materials were thoroughly mixed and loaded into quartz crucibles, which were then sealed in evacuated quartz ampoules under a vacuum of approximately 10^−3^ Pa. The ampoules were first heated to 1273 K within 1 h and maintained at this temperature for 24 h to ensure complete reaction and homogenization. Subsequently, the temperature was rapidly decreased to 1153 K within 1 h, followed by slow cooling to 1053 K over 200 h to promote crystal growth [29]. To accommodate the lower melting point of Ni and suppress volatilization, the soaking temperature was gradually decreased with increasing Ni content, while the overall heating and cooling procedure was kept similar. After furnace cooling to room temperature, the obtained crystals were collected and separated from excess flux by centrifugation at 36,000 rpm. The as-grown samples were then ground into fine powders for subsequent characterization and electrochemical measurements. Each composition was synthesized at least twice to ensure reproducibility.

### 2.2. Structural and Compositional Characterization

The crystal structures of the samples were characterized by single-crystal X-ray diffraction (XRD) by a Bruker D8 (Bruker Corporation, Karlsruhe, Germany). Advance diffractometer with Cu Kα radiation (λ = 1.5406 Å) over a 2θ range of 10–90° with a step size of 0.0156°. The actual elemental compositions of the samples were determined by energy-dispersive X-ray spectroscopy (EDS) on an FEI Nova NanoSEM 450 (FEI, Brno, Czech Republic) scanning electron microscope operating at an accelerating voltage of 10 kV. EDS spot analysis was used to quantify the Ni content across all five compositions, while elemental mapping was additionally performed on the *x* = 0.6 sample to evaluate the spatial distribution of constituent elements. The measured compositions were found to be very close to the nominal doping levels. The samples are labeled according to their nominal compositions, while composition-dependent discussions are based on the actual Ni contents determined by EDS.

### 2.3. Electrochemical Measurements

The HER electrocatalytic performance was evaluated in a standard three-electrode configuration using 1.0 M KOH as the electrolyte. A glassy carbon electrode was used as the working electrode, Hg/HgO as the reference electrode, and a Pt foil as the counter electrode.

The catalyst ink was prepared by dispersing 10 mg of catalyst powder in a mixed solvent containing 480 μL ethanol, 480 μL deionized water, and 40 μL 5 wt% Nafion, followed by ultrasonic treatment to obtain a homogeneous suspension. The ink was drop-cast onto the glassy carbon electrode (geometric area ≈ 0.07 cm^2^) in two successive aliquots of 5 μL each, corresponding to a catalyst mass loading of 1.43 mg/cm^2^.

Linear sweep voltammetry (LSV) was carried out at a scan rate of 5 mV s^−1^, and all measured potentials were converted to the reversible hydrogen electrode (*RHE*) scale according to

ERHE=EHgHgO+0.098+0.059×pH

with 90% iR compensation applied during data processing. Tafel slopes were derived from the polarization curves. The electrochemical impedance spectroscopy (EIS) was performed at a potential of −1.5 V (vs. *Hg*/*HgO*) over a frequency range of 100 kHz to 0.1 Hz with an AC perturbation amplitude of 5 mV.

The double-layer capacitance ($C_{dl}$) was estimated from cyclic voltammetry conducted in the non-faradaic region at scan rates of 20, 40, 60, 80, 100, and 120 mV s^−1^. The $C_{dl}$ values were obtained from half of the difference between the anodic and cathodic current densities (ΔJ/2) at a fixed potential plotted against the scan rate.

### 2.4. First-Principles Calculations

All first-principles calculations were performed using the Vienna Ab initio Simulation Package (VASP, version 5.4.4) [30,31] within the framework of density functional theory (DFT). The projector augmented wave (PAW) [32,33] method was used to describe the electron–ion interactions, and the exchange-correlation functional was treated within the generalized gradient approximation (GGA) in the form of Perdew–Burke–Ernzerhof (PBE) [34]. The plane-wave cutoff energy was set to 400 eV, and a Gamma-centered 5 × 5 × 1 Monkhorst-Pack k-point mesh was used for Brillouin zone sampling of the 2 × 2 × 1 supercell containing 48 atoms. Spin polarization (ISPIN = 2) was enabled throughout all calculations to account for the magnetic character of the Fe/Ni sublattices, with initial magnetic moments of 3 μ*_B_* assigned to each transition-metal atom.

Structural optimization was first carried out for models with different Ni doping concentrations, allowing the lattice parameters and atomic positions to fully relax (ISIF = 3). The convergence criteria were set to 1 × 10^−4^ eV for the total energy and 0.02 eV/Å for the Hellmann–Feynman forces. Gaussian smearing (ISMEAR = 0) with a broadening of σ = 0.15 eV was applied during structural relaxation. Subsequent static self-consistent calculations were performed with a tighter energy convergence of 1 × 10^−6^ eV and a reduced smearing parameter of σ = 0.05 eV to obtain reliable electronic-structure information. For the density of states calculations, the tetrahedron method with Blöchl corrections (ISMEAR = −5) was adopted, and the charge density was read from the static self-consistent step (ICHARG = 11). The projected density of states (PDOS) was extracted using LORBIT = 11, and the *d*-band center was derived from the projected d-orbital density of states of the transition-metal sites according to

$$\varepsilon_{d}=\frac{\int E\cdot \rho_{d}\left(E\right)dE}{\int \rho_{d}\left(E\right)dE}$$

where $\rho_{d}\left(E\right)$ denotes the projected d-orbital density of states.

For the experimentally optimal composition (*x* = 0.6), the valence charge density was further computed on the relaxed bulk structure to visualize the electron-enriched regions associated with the transition-metal sites. A dense FFT grid of 126 × 126 × 336 was adopted to ensure high spatial resolution of the charge-density distribution. Charge-density maps were generated and visualized using VESTA [35].

## 3. Structural and Compositional Analysis of (Fe_1−x_Ni_x_)_3_GaTe_2_ Samples

Figure 1b shows the single-crystal XRD patterns of the (Fe_1−*x*_Ni*_x_*)_3_GaTe_2_ samples. All observed reflections can be indexed to the space group P6_3_/mmc [19,29], and no additional diffraction peaks attributable to impurity phases are detected within the investigated composition range, indicating that Ni incorporation does not change the parent crystalline structure. The sharp diffraction features further suggest the high quality of the as-grown samples obtained by the self-flux method.

To better evaluate the structural evolution induced by Ni doping, the diffraction peaks (006) in the 31–36° range are enlarged in the inset of Figure 1b. With increasing Ni content, the characteristic diffraction peaks exhibit an overall shift toward higher diffraction angles. Notably, this shift is more evident in the doping range from *x* = 0.2 to *x* = 0.6, whereas only a slight shift is observed from *x* = 0.6 to *x* = 0.8, and almost no further shift can be identified from *x* = 0.8 to *x* = 1.0. This trend agrees with previous reports on the same system [19,36].

Based on the SEM images (Figure S1) and optical microscope images (Figure S2), the crystals exhibit high crystallinity and excellent morphological uniformity. The SEM images show flat, smooth, and crack-free surfaces with sharp edges and clear layered steps, indicating a well-preserved layered structure. Optical microscope images further confirm the presence of large and smooth pieces. Both characterization techniques consistently reveal a plate-like morphology with clean and distinct grain boundaries across all *x* values. These results demonstrate that substitution does not noticeably deteriorate the crystal quality.

EDS analysis (Figure 1c) detects clear Fe, Ga, Te, and Ni signals across the series. Elemental mapping of the *x* = 0.6 sample (Figure 1d–g) further shows uniform distribution of all four elements without segregation.

## 4. Electrocatalytic HER Performance and Electrochemical Kinetics in Alkaline Media

**Figure 2:**
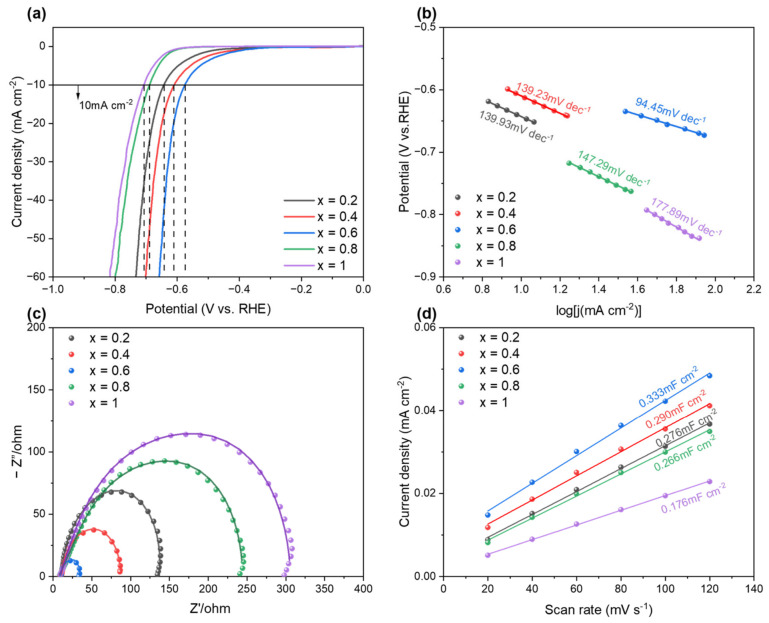
**Electrocatalytic HER performance of (Fe_1−*x*_Ni*_x_*)_3_GaTe_2_ in 1.0 M KOH.** (**a**) LSV curves of samples with different Ni contents. (**b**) Corresponding Tafel plots derived from the polarization curves. (**c**) Nyquist plots obtained from electrochemical impedance spectroscopy (EIS). (**d**) Double-layer capacitance ($C_{dl}$) analysis based on cyclic voltammetry in the non-faradaic region.

The HER performance of the (Fe_1-*x*_Ni*_x_*)_3_GaTe_2_ series was evaluated in 1.0 M KOH using a standard three-electrode configuration. The iR-corrected linear sweep voltammograms (LSVs) are presented in Figure 2a. At a benchmark current density of 10 mA cm^−2^ [37,38], the overpotentials are 644, 611, 576, 689 and 708 mV for *x* = 0.2, 0.4, 0.6, 0.8 and 1.0, respectively, showing a non-monotonic, volcano-shaped composition dependence [39,40]. This trend suggests that Ni primarily modulates the host framework rather than acting as an independent active site.

Kinetic insight was obtained from the Tafel plots derived from the polarization curves (Figure 2b). The Tafel slopes are 140, 139, 94, 147 and 178 mV dec^−1^ for *x* = 0.2, 0.4, 0.6, 0.8 and 1.0, respectively. Electrochemical impedance spectroscopy (EIS) was further employed to investigate the interfacial kinetics. The Nyquist plots in Figure 2c show that all samples exhibit a single depressed semicircle in the high-frequency region, with (Fe_0.4_Ni_0.6_)_3_GaTe_2_ showing the smallest semicircle and hence the lowest charge-transfer resistance.

The electrochemically active surface area (ECSA) was probed via the double-layer capacitance ($C_{dl}$), extracted from cyclic voltammograms recorded in the non-faradaic region at varying scan rates [37]. Cyclic voltammograms of (Fe_1-*x*_Ni*_x_*)_3_GaTe_2_ recorded at various scan rates from 20 to 120 mV s^−1^ are shown in Figure S3 (Supporting Information). As shown in Figure 2d, plotting the half-difference of anodic and cathodic current densities (ΔJ/2) against the scan rate yields a linear response for every composition, and the slope of this linear fit directly gives $C_{dl}$. The extracted $C_{dl}$ values are 0.276, 0.290, 0.333, 0.266 and 0.176 mF cm^−2^ for *x* = 0.2, 0.4, 0.6, 0.8 and 1.0, respectively. The maximum at *x* = 0.6 indicates that a greater fraction of catalytic sites is electrochemically wetted and contributes to the faradaic process at the optimal composition. Moreover, long-term durability is a critical test for the practical application of electrocatalysts in water splitting. The long-term durability of the (Fe_1-*x*_Ni*_x_*)_3_GaTe_2_ was further evaluated by chronoamperometric (i-t) measurements. As shown in Figure S4 (Supporting Information), (Fe_0.4_Ni_0.6_)_3_GaTe_2_ exhibits the most outstanding stability among all the synthesized catalysts, with a current retention of 84.27% after 10 h of continuous operation. The four electrochemical descriptors—overpotential, Tafel slope, R_ct_ and $C_{dl}$—therefore consistently identify *x* = 0.6 as the optimum, pointing to an underlying electronic-structure origin that we examine next by first-principles calculations.

## 5. Electronic-Structure Evolution Induced by Ni Doping

**Figure 3:**
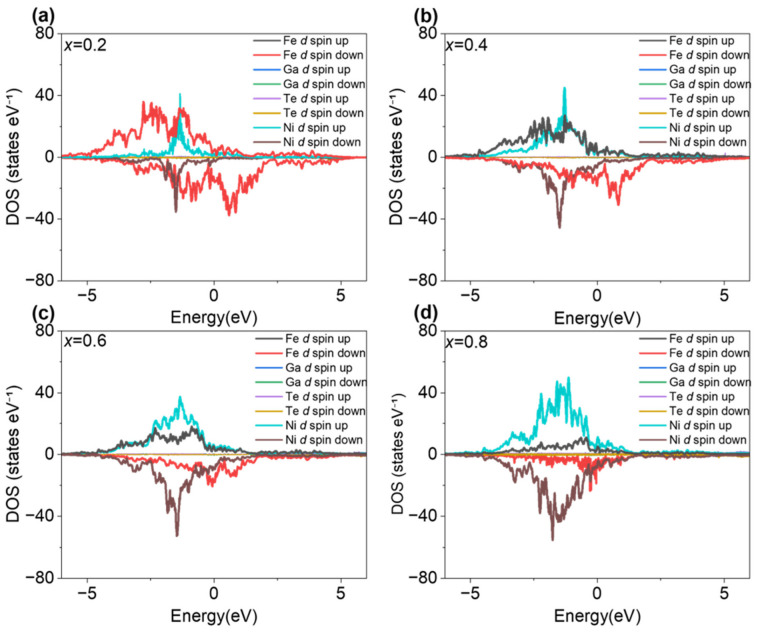
**Projected density of states (PDOS) of (Fe_1−*x*_Ni*_x_*)_3_GaTe_2_ with different Ni contents.** (**a**) *x* = 0.2, (**b**) *x* = 0.4, (**c**) *x* = 0.6, (**d**) *x* = 0.8. The electronic states near the Fermi level are mainly dominated by Fe/Ni *d* orbitals.

To clarify the electronic-structure origin of the doping-dependent HER behavior, spin-polarized projected density of states (PDOS) calculations were performed for (Fe_1−*x*_Ni*_x_*)_3_GaTe_2_ across the experimentally accessed composition range. As shown in Figure 3, the electronic states near the Fermi level (E_F_) are dominated by the *d* orbitals of Fe and Ni, with negligible contributions from Ga and Te [41]. As Norskov and co-workers have shown, the hydrogen binding strength on a metal surface is often linked to its *d*-band center [42]. They also showed that the further the *d*-band center is from the Fermi level, the weaker the hydrogen binding [43]. However, this trend-based correlation does not quantitatively determine the absolute value of ${\Delta G}_{H^{*}}$. Thus, the *d*-band center is a useful indicator but not a direct substitute for ${\Delta G}_{H^{*}}$. Accordingly, we focus on the *d*-band center to rationalize the observed kinetic trends for comparing relative HER activities among different compositions.

**Figure 4:**
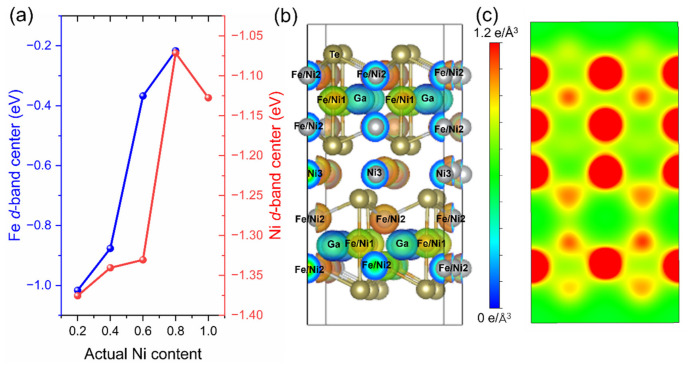
**Electronic-structure analysis of (Fe_1−*x*_Ni*_x_*)_3_GaTe_2_.** (**a**) The *d*-band centers of Fe (blue, left axis) and Ni (red, right axis) as a function of the actual Ni content *x*, referenced to the Fermi level (E_F_ = 0 eV). (**b**) Three-dimensional valence charge-density isosurface (level = 0.1 e/Å^3^) of (Fe_0.4_Ni_0.6_)_3_GaTe_2_, showing the spatial distribution of valence electrons around the three transition-metal Wyckoff sites (intralayer Fe/Ni1 and Fe/Ni2, interlayer Ni3) together with the Ga and Te sublattices. The labels indicate the corresponding sites for cross-reference with panel (**c**). (**c**) Cross-sectional valence charge-density map of (Fe_0.4_Ni_0.6_)_3_GaTe_2_ on the (1 0 0) plane near the origin, showing a B-G-R color scale ranging from 0 to 1.2 e/Å^3^. Red regions indicate higher valence charge density, predominantly localized on the Fe and interlayer Ni3 sublattices that contribute the dominant *d*-states near E_F_.

To quantify this redistribution, the *d*-band centers (ε*_d_*) of the Fe and Ni sites were extracted as a function of Ni content (Figure 4a) [44,45]. With increasing *x*, the Fe *d*-band center exhibits a pronounced upward shift from −1.017 eV at *x* = 0.2 to −0.217 eV at *x* = 0.8, while the Ni *d*-band center remains comparatively confined within a narrow window between −1.376 and −1.072 eV over the same range. The most striking feature is an abrupt jump of approximately 0.5 eV in the Fe *d*-band center between *x* = 0.4 (−0.877 eV) and *x* = 0.6 (−0.368 eV), which coincides with the experimentally observed activity maximum at (Fe_0.4_Ni_0.6_)_3_GaTe_2_. Beyond this composition, the Fe *d*-band center continues to shift upward but at a markedly reduced rate, and the HER activity decreases rather than improves further.

These results suggest two key points. First, the comparatively stable Ni ε*_d_* together with the strongly composition-dependent Fe ε*_d_* suggests that Ni acts predominantly as a modulator of the Fe-site electronic environment rather than as the dominant active site itself, in agreement with the site-occupancy road map of (Fe_1-*x*_Ni*_x_*)_3_GaTe_2_, in which Ni preferentially occupies the interlayer Ni3 site and the intralayer Fe2 site at intermediate compositions while the Fe1 sites remain Fe-occupied up to *x* ≈ 0.75 [19]. Second, the abrupt Fe ε*_d_* upshift between *x* = 0.4 and *x* = 0.6 likely marks a key composition window in which the local coordination of the Fe sublattice, achieved through Ni filling of the adjacent Fe2 and interlayer Ni3 positions, brings the Fe *d* band closest to the optimal hydrogen-binding regime described by the volcano principle [39,40]. Further Ni incorporation pushes ε*_d_* past this optimum and weakens the M-H interaction excessively [46], accounting for the observed activity decline despite continued electronic-state evolution. This explains why *x* = 0.6, rather than the most upshifted composition, sits at the volcano apex.

## 6. Charge-Density Analysis of the Optimal Composition

To identify which regions of (Fe_0.4_Ni_0.6_)_3_GaTe_2_ are electronically favorable for adsorbing H* and water-derived intermediates, we computed the valence charge density on its relaxed structure. The charge density was projected along the a-axis (Figure 4c) so that the Fe1/Fe2 layers and all four interlayer Ni3 atoms appear in the same plane, allowing intralayer and interlayer Ni contributions to be discriminated.

The charge-density map (Figure 4c) reveals a clear spatial differentiation of the valence electron distribution. The regions surrounding the Fe sublattice and the interlayer Ni3 sites display markedly higher charge density, whereas the Ga sublattice exhibits relatively lower values. The Fe and interlayer Ni3 regions are therefore identified as the dominant electron-localization regions of (Fe_0.4_Ni_0.6_)_3_GaTe_2_, in spatial agreement with the sublattices that contribute the dominant d-states near E_F_ [47].

This spatial pattern is fully consistent with the electronic-structure picture established above. The Fe sublattice and the interlayer Ni3 sites are precisely the positions that contribute the dominant *d*-band states near EF (Figure 3), and their Fe and Ni *d* band account for the locally enhanced valence charge density at these sites. The Fe and interlayer Ni3 sublattices are thus simultaneously favored by both the *d*-band and charge-density criteria at *x* = 0.6.

## 7. Conclusions

In summary, we demonstrate that the alkaline HER activity of layered (Fe_1-*x*_Ni*_x_*)_3_GaTe_2_ is controlled by a site-specific upshift of the Fe-sublattice *d*-band center rather than by the Ni content itself. The activity peaks at *x* = 0.6, where a rapid upshift of the Fe *d*-band center coincides with locally enhanced valence charge density on the Fe and interlayer Ni3 sites. Ni therefore acts as a modulator of the Fe environment rather than as the dominant active site. The same composition–occupancy–activity analysis framework is not limited to this system but can be extended to other multi-Wyckoff-site van der Waals magnets.

## Data Availability

The original contributions presented in this study are included in the article/Supplementary Material. Further inquiries can be directed to the corresponding authors.

## Supplementary Materials

The following supporting information can be downloaded at https://www.mdpi.com/article/10.3390/nano16130820/s1, Figure S1: **Scanning electron microscope image of (Fe_1−*x*_Ni*_x_*)_3_GaTe_2_.** (a) *x* = 0.2; (b) *x* = 0.4; (c) *x* = 0.6; (d) *x* = 0.8; (e) *x* = 1. Figure S2: **Optical microscope image of (Fe_1−*x*_Ni*_x_*)_3_GaTe_2_.** (a) *x* = 0.2; (b) *x* = 0.4; (c) *x* = 0.6; (d) *x* = 0.8; (e) *x* = 1. Figure S3: **CV curves of (Fe_1−*x*_Ni*_x_*)_3_GaTe_2_ in the non Faradaic potential portion at various scan rates in 1.0 M KOH electrolyte.** (a) *x* = 0.2; (b) *x* = 0.4; (c) *x* = 0.6; (d) *x* = 0.8; (e) *x* = 1. Figure S4: **Long-term durability of (Fe_1−*x*_Ni*_x_*)_3_GaTe_2_ in 1.0 M KOH electrolyte.**
