# Supplementary material for: d-Band Engineering of Layered (Fe1−xNix)3GaTe2 for Enhanced Alkaline Hydrogen Evolution by Ni-Substitutional Doping
Source: Nanomaterials (Basel). 2026 Jul 2;16(13):820. doi: 10.3390/nano16130820 (PMC13362656; doi:10.3390/nano16130820)
Supplement: Supplementary file 1 [file nanomaterials-16-00820-s001.zip › nanomaterials-4329349-supplementary.pdf]

Supplementary Information for

d-Band Engineering of Layered $(Fe_{1-x}Ni_x)_3GaTe_2$ for Enhanced Alkaline Hydrogen Evolution by Ni-Substitutional doping

Xiaomin Tian[1,†], Yuan Cao [1,†], Huilin Zhou[1,†], Fanjie Tan[1], Ziqing Zhang[1], Liying Pei[1], Yi Ma[2*], Jianzhi Gao[1], Wenliang Zhu[1*] and Minghu Pan[1*]

[1]School of Physics and Information Technology, Shaanxi Normal University, Xi'an 710062, China

[2]School of Chemistry and Chemical Engineering, Shaanxi Normal University, Xi'an 710062, China

[†]These authors contribute equally to this work.
*Corresponding authors' E-mail:
wlzhu@snnu.edu.cn (W. Zhu): 0000-0002-7870-8530;
mayi@snnu.edu.cn (Y. Ma): 0000-0001-8143-1483;
minghupan@snnu.edu.cn (M. Pan): 0000-0002-1520-209X.

Experimental Section

The surface morphologies of the samples were characterized by scanning electron microscopy (SEM) on an FEI Nova NanoSEM 450.

Cyclic voltammetry (CV) measurements were conducted in the non-faradaic potential region to evaluate the electrochemical double-layer capacitance (C_{dl}) of the $(Fe_{1-x}Ni_x)_3GaTe_2$ electrode. The potential window was selected as -0.6 - -0.7 V *vs.* Hg/HgO to ensure only non-faradaic charging currents were recorded.

The long-term durability of the $(Fe_{1-x}Ni_x)_3GaTe_2$ electrode toward the hydrogen evolution reaction (HER) was evaluated by chronoamperometric (i-t) measurement. The i-t test was performed at a constant applied potential corresponding to a current density of 10 mA cm^{-2} for 10h without iR compensation. Prior to the stability test, the

working electrode was subjected to several cyclic voltammetry cycles until a stable response was achieved.

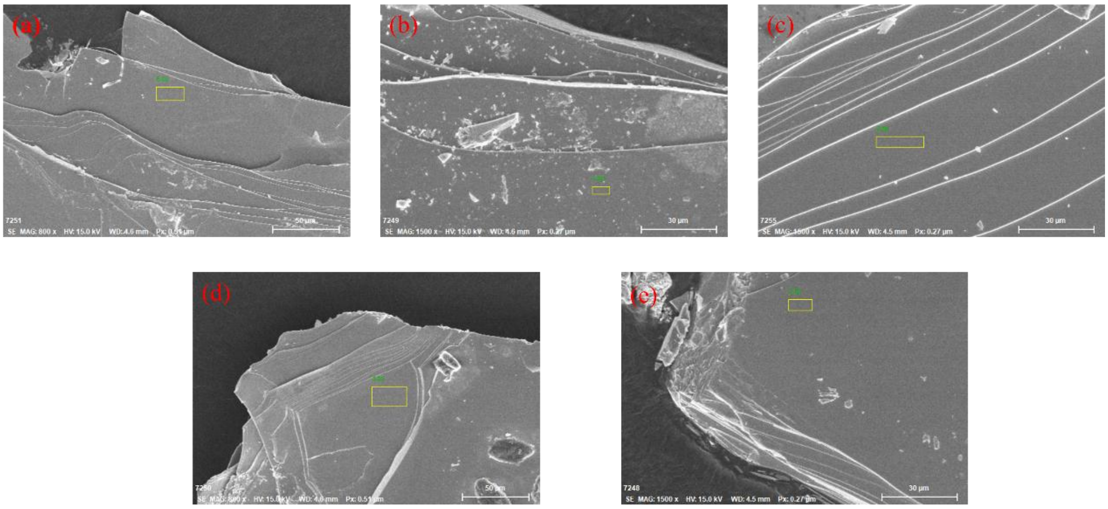

Figure S1. Scanning electron microscope image of $(Fe_{1-x}Ni_x)_3GaTe_2$. (a) x=0.2; (b) x=0.4; (c) x=0.6; (d) x=0.8; (e) x=1.

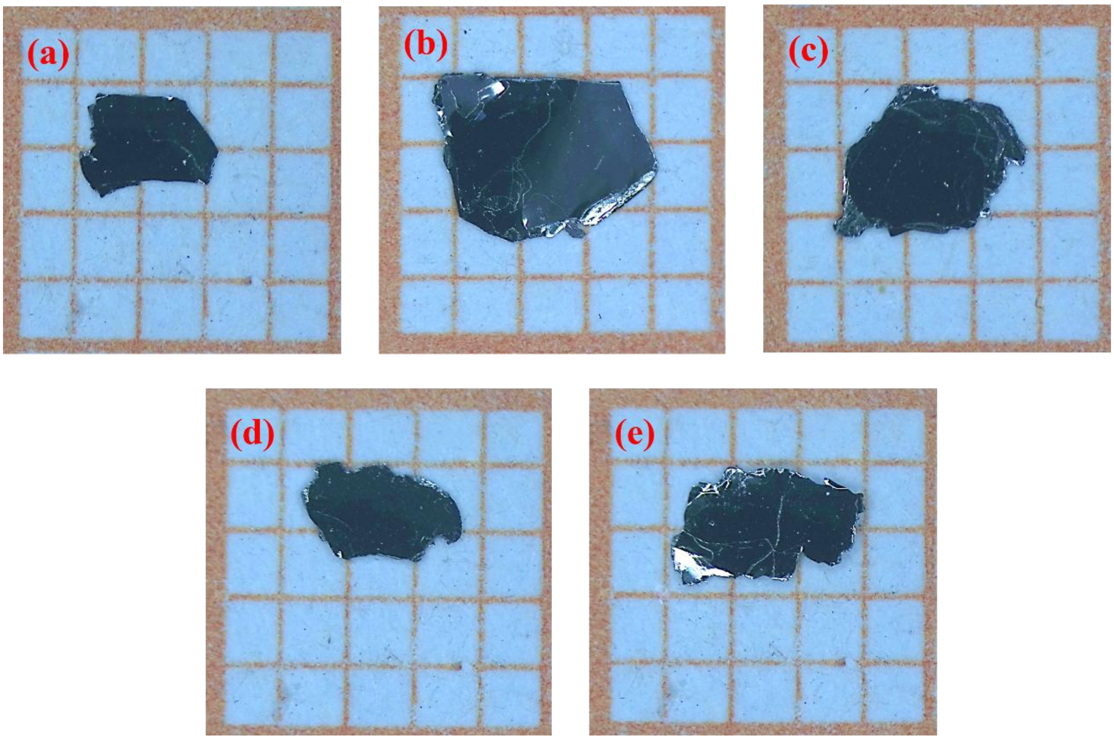

Figure S2. Optical microscope image of $(Fe_{1-x}Ni_x)_3GaTe_2$. (a) x=0.2; (b) x=0.4; (c) x=0.6; (d) x=0.8; (e) x=1.

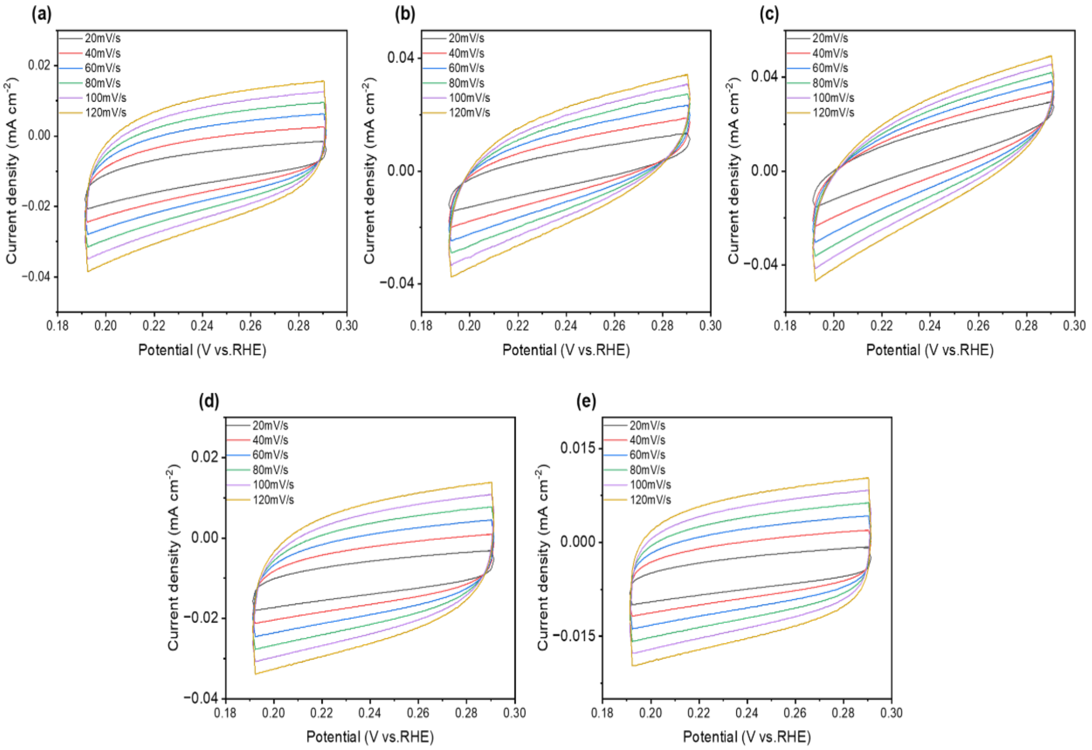

Figure S3. CV curves of $(Fe_{1-x}Ni_x)_3GaTe_2$ in the non-faradaic potential portion at various scan rates in 1.0 M KOH electrolyte. (a) x=0.2; (b) x=0.4; (c) x=0.6; (d) x=0.8; (e) x=1.

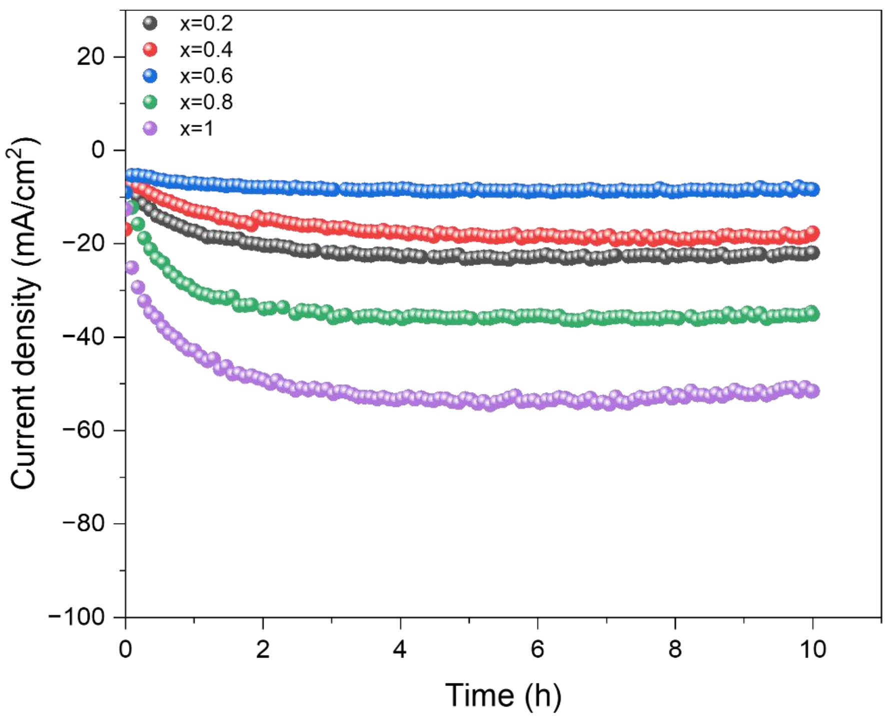

Figure S4. Long-term durability of $(Fe_{1-x}Ni_x)_3GaTe_2$ in 1.0 M KOH electrolyte
